# Objectively measured physical activity in population-representative parent-child pairs: parental modelling matters and is context-specific

**DOI:** 10.1186/s12889-018-5949-9

**Published:** 2018-08-17

**Authors:** Bettina Bringolf-Isler, Christian Schindler, Bengt Kayser, L. Suzanne Suggs, Nicole Probst-Hensch, the SOPHYA Study Group, Nadja Mahler, Urs Mäder, Thomas Wyss, Nadine Stoffel, Kathrin Favero, Andrea Poffet, Jvo Schneider, Lisa Guggenbühl, Charlotte Braun-Fahrländer, Cornelis de Hoogh, Simone Isler

**Affiliations:** 1 Swiss Tropical and Public Health Institute, Socinstrasse 57, 4051 Basel, Switzerland; 2 University of Basel, Petersplatz 1, 4051 Basel, Switzerland; 3 Institute of Sport Sciences, University of Lausanne, 1015 Lausanne, Switzerland; 4 Institute for Public Communication, Università della Svizzera Italiana, Via G. Buffi 13, 6900 Lugano, Switzerland

**Keywords:** Accelerometer, Children, Adolescents, Co-activity, Cycling, Sociodemographics, Walkability, Language region

## Abstract

**Background:**

Evidence for the context-specific influence of parental modelling on physical activity (PA) in childhood remains inconclusive. This nationwide Swiss study assessed the cross-sectional association between objectively measured PA of parents and their children and whether it varied across different levels of sociodemographic and environmental factors. In a second step a structural equation-model (SEM) was used to assess, whether associations between children’s PA and sociodemographic and environmental factors are mediated by the parental PA behaviour.

**Methods:**

The population-based sample of the SOPHYA-study consisted of 889 children aged 6 to 16 years living in Switzerland and 1059 parents. PA was measured using accelerometers. Information on sociodemographics, sports behaviour, family characteristics, and perceived environment was obtained by telephone interview and parental questionnaire. Objective environmental data was allocated to each family’s residential address using GIS (geographic information system). A structural equation model tested these factors for both independent associations with children’s PA and associations mediated through the parental PA behaviour.

**Results:**

Parental moderate to vigorous physical activity (MVPA) was associated with MVPA of their children in general (*p* < 0.001). Correlations between parents’ and children’s MVPA were stronger for children aged 10–12 years and for those living in the Italian speaking part of Switzerland. An increase of 1 min of mother’s and of father’s MVPA was associated with 0.24 and 0.21 min more MVPA in children, respectively. Father’s PA was associated with that of their sons, but not with that of their daughters, whereas the association of mothers’ and children’s PA did not depend on the parent-offspring sex-match. The pathway analysis in our structural equation model showed direct effects on children’s MVPA as well as indirect effects mediated by the parental PA behaviour.

**Conclusions:**

Parental modelling seems relevant for children’s PA, but not to the same degree in all children. Interventions focusing on strengthening parental PA behaviour for the promotion of PA in the young must consider additional contextual factors related to the socio-cultural and structural environment.

**Electronic supplementary material:**

The online version of this article (10.1186/s12889-018-5949-9) contains supplementary material, which is available to authorized users.

## Background

Physically active children are leaner, exhibit better cardiovascular profiles, and do better with regard to psychological and social well-being [1–3]. Yet, on a global scale children’s physical activity (PA) levels are decreasing [4]. Childhood and adolescence represent important life stages for health promotion because the behavioural patterns established during these years tend to track into adulthood [5].

For the design of effective PA promoting strategies it is important to understand factors contributing to children’s active lifestyle. According to the ecological model PA depends on different levels including the individual behaviour, the social and physical environments, as well as policies and all these levels interact with each other. So far factors of the macro environment (neighbourhood) as well as the microenvironment (families) have more often been analysed separately than taking into consideration their complex interrelations [6]. Limited evidence suggests that the family environment may be more influential on children’s PA than the neighbourhood environment [7]. Parents are most often the main decision makers [8] and may promote an active lifestyle in their children through role modelling.

Role modelling has been described as a form of influence whereby children reproduce the behaviours of their parents through observational and social learning processes [9]. Studies testing this hypothesis in the PA domain found mixed results [10–12], in part due to methodological limitations [12]. First, most studies are based on self- and proxy reports of PA, which is sensitive to social desirability bias and not recommended for the assessment of children’s overall PA [13]. Associations between parents and children’s PA could be the result of correlated reporting bias of parents’ own and children’s PA. Yet, Yao et al. [12] showed that PA associations were stronger if measured objectively. Except for the studies from Jago et al. [14] and Stearns [15], objective methods of both parent and child PA were mostly applied to small samples with limited generalizability of the findings [11]. Second, several studies investigated parent-child PA associations without distinguishing fathers’ and mothers’ PA [12]. Time co-spent and role model function may vary by the sex match of parent-child pairs [15]. A third and important limitation is the sparse assessment of modifiers and mediators of the parent-child PA relationship [16]. As an example one could expect that the parent-child PA relationship decreases by age while in parallel the influence of peers becomes more important. How PA associations between parents and children depend on children’s age [14], socio-economic status (SES), and cultural or geographic context remains poorly understood. As Brockmann et al. showed [17], children from low SES tend to have less access to structured exercise, but engage more in unstructured play. How much children engage in organized versus non-organized PA differs by region [18]. PA associations between parents and children may also differ by the workload of the parents, family cohesion or family factors such as having siblings [19]. The aim of the present study was to assess whether there is an association between children’s and parental PA (overall and separately for mother’s or father’s PA) and whether it varies across different levels of the ecological model (sociodemographics and environmental factors). To test such differences across the levels of the ecological model are important for providing insights into whether family-based interventions might be effective for specific subgroups or for everyone [15, 16]. In a second step a structural equation model (SEM) was used to assess, whether associations between children’s PA and factors representing different levels of the ecological model are mediated by the parental physical activity behaviour.

## Methods

The data used in this paper come from the nationwide SOPHYA sample [20, 21]. Analyses test the associations between objectively measured PA of parents and their children aged 6 to 16 years old and the modification by age, sex, family, sociocultural and environmental factors.

### Study population

**Fig. 1:**
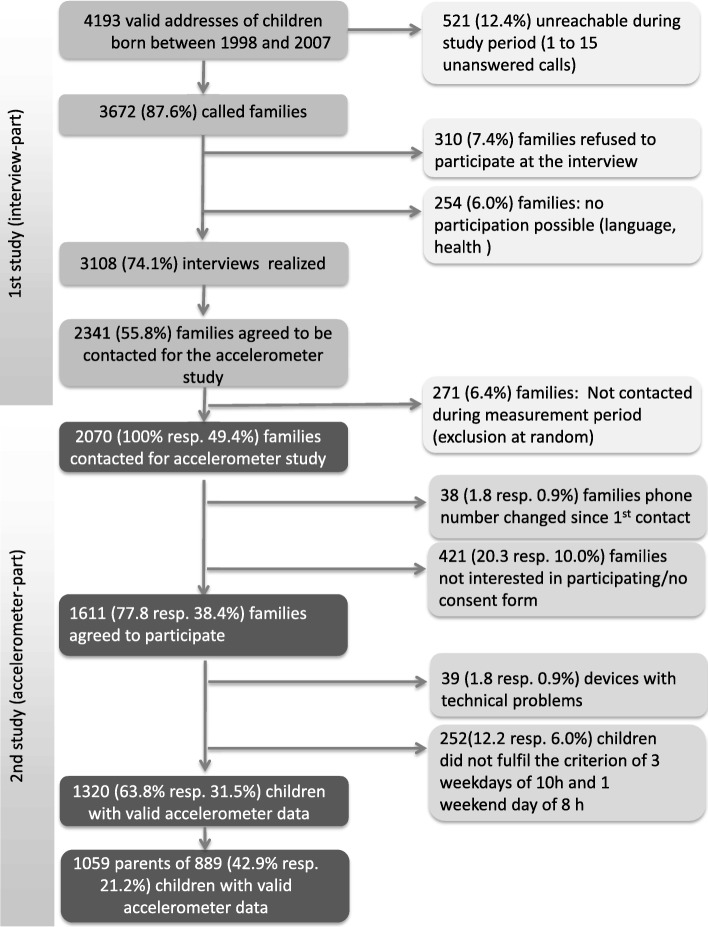
Flow chart of the study population

The recruitment of the study sample was based on national population register data. Children were eligible if they were registered in Switzerland and born between 1998 and 2007. A stratified sampling procedure (10 equivalent strata, one per year) was applied allowing for balanced age groups. The study consisted of two phases: First, children were recruited for a telephone interview about sport behaviour between 2013 and 2015. At the end of the interview the families were asked whether they could be contacted for a second study using accelerometers. Second, a random sample of agreeing families were re-contacted between 2014 and 2015 by trained fieldworkers and asked whether the child and at least one parent would wear an accelerometer for one week. If the families agreed, accelerometers and instructions for their use were sent to the participants by mail after an oral instruction by phone. All instruments could be sent back to the study center with a prepaid etiquette. In the present analysis, families were included if they provided valid accelerometer measurements of the child and of at least one parent resulting in a sample of 889 children and 1059 parents (Fig. 1). Families did not get any compensation for their participation. The study protocol was approved by the ethics committee of the Canton Basel (EKBB). All parents and children aged 12 years and older gave written informed consent for their participation. For the younger children a parent provided the written informed consent.

### Data collection

#### Telephone interview at baseline

The telephone interview was conducted by a professional field research institute (LINK institute, Lucerne, Switzerland) using computer-aided telephone interviews (CATI). Families were first sent a letter explaining the study aim and announcing the phone call. The main focus of the interview was on sport participation of the child at school and during leisure time (type of sport; duration; location; social context). This allowed distinguishing between families in which children regularly performed sport with their parents versus families with no regular parental sport co-activities. The interview additionally provided information on the socioeconomic status (SES) related factors (age, sex, nationality and parental education). Interviews were conducted in one of three national languages (German, French, Italian). Children aged 11 years and older were interviewed in the context of the study “Sport Schweiz” [22], for younger children (10 years and younger) a proxy interview was conducted.

#### Accelerometry

Children’s and parent’s physical activity was measured using Actigraph accelerometers models GT1M and GT3X (ActiGraph, Pensacola, Florida). The measurements took place between December 2013 and June 2015. Children and parents were asked to wear the accelerometers on the hip for seven consecutive days. The devices were removed for water activities and during sleeping hours [23]. The initialisation of the devices, the download of the data and the data reduction were conducted using the ActiLife software (ActliLife 6.12, Pensacola, Florida). Epoch time was set at 15 s. A period of more than 60 min of consecutive zeros was defined as non-wearing time. Participants were included in the analyses if they provided measurements of ≥10 h on at least three weekdays and of ≥8 h on at least one weekend day [24]. Mean daily physical activity was calculated by multiplying the mean on weekdays by 5 and the mean of the weekend day(s) by 2 and dividing the sum by 7, in order to account for the different physical activity patterns on weekdays and in the weekend. To define children’s time spent in moderate to vigorous physical activity (MVPA) the age-dependent cut-offs of Freedson [25] with a threshold of 4 metabolic equivalents was used. For parents a cut-off of 2020 counts per minute was applied [26]. Both total MVPA time and MVPA time using bouts of 10 consecutive active minutes (with a tolerance range of 2 min) were calculated for parents.

#### Hard-copy questionnaire during physical activity measurement week

A parental and a children questionnaire, both completed by the parents during the measurement week, included questions on specific aspects of parent’s and children’s physical activity during the measurement week (e.g. time spent cycling in hours/week or the number of times they went to a sport club per week- both representing parental modelling on domain specific PA) and on their weight and height, workload of the parents and number of siblings of the child. Children’s quality of life was assessed using the validated KINDL^R^ questionnaire [27]. This questionnaire consists of six different modules (four questions each) which can be combined to a summary score or used as single outcome. For the present analyses the score of the module focusing on the family wellbeing was used to map the parent-child relationship (each question consisting on a 5 point item from never to “all the time”). The family’s wellbeing was assessed with following four questions: “my child got along well with us as parents”, “my child felt fine at home”,“we quarrelled at home”, and “my child felt that a parent was bossing him/her around”.

#### Environmental factors

Families were asked to indicate their home address for deriving a residential walkability index, a validated measure of how pedestrian friendly an area is [28]. It was calculated for a 1 km radius buffer and is based on three components: the residential density (households per residential acre), the intersection density (number of road intersections per 1 km-buffer) and the land use mix. All spatial analyses were conducted with ArcGIS 10.2.1(ESRI). The calculation of residential and intersection density was based on census data [29] and the road network from Swiss Topo Vector 25 [30]. The land use mix of the built environment derived from Corine land cover data base [31], which differentiates between “residential”, “industrial or commercial” and “entertainment” units. Corine has been used to make the data comparable for the whole country and even for Europe. The language region and the degree of urbanity were defined according to the classifications and the boundaries of the Swiss Federal Statistical Office [32].

### Data analysis

**Fig. 2:**
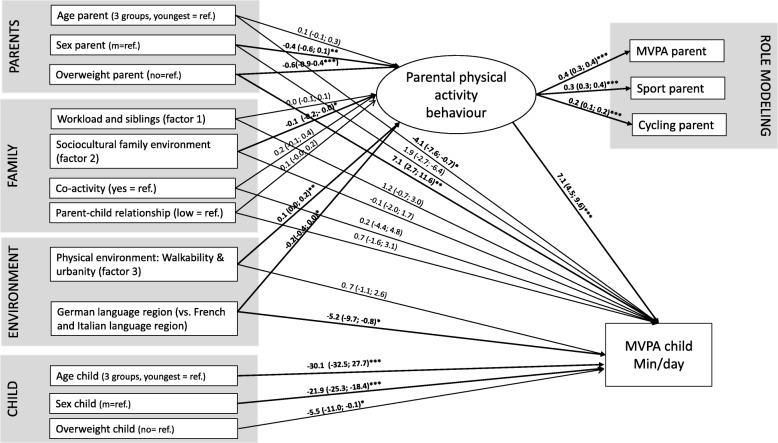
SEM of the direct and the indirect (via parental PA behavior) effect on children’s MVPA, SEM = structure equation model, Family factor 2 is based on nationality (non-Swiss = reference) and parental education (low = reference), The values display the average change (Coeff. (95% Confidence interval)) in the child’s minutes spent in MVPA/day associated with the respective factor level (as compared to the reference level) or with a one unit change in the respective predictor variable, * *p* < 0.05, ** *p* ≤ 0.01, *** *p* ≤ 0.001, *N* = 852, if both parents wore an accelerometer the mother has been included. Fit statistic: RMSEA (root mean square error of approximation): 0.04 (90%CI: 0.03; 0.05), NNFI (non-normed fit index): 0.904, CFI (comparative fit index) 0.947. R^2^ of the model for the latent variable “parental physical activity behaviour” = 0.123

Differences in children’s and parent’s characteristics respectively were assessed using the Kruskal Wallis-test. To test the association between parental (independent variable) and children’s MVPA (dependent variable and main outcome), linear regression models adjusting for age, age^2^, sex of the child, measurement season, accelerometer type (GT3x or GT1M - although no effect) and accelerometer time were conducted. To calculate the mean increase of children’s MVPA per minute increase of their mothers and fathers MVPA respectively, the regression estimates were determined using a bootstrap with 1000 replications. For all other regression models MVPA measurements of children and parents were log-transformed to achieve a close to normal distribution of residuals. Separate models were run by sex of the parent and for parent’s total MVPA time and for parent’s MVPA time using 10 min bouts only. In a next step the models were stratified by possible moderators (age of the child, sex of the child, family characteristics, PA role modelling of the parents, and environmental factors as listed in Table 1). Moderators are variables that affect the direction and or the strength of the relation between an independent variable and a dependent variable [33]. Differences in effect estimates across strata were assessed by a chi2-test for heterogeneity. The presence of significant heterogeneity was interpreted as indicating moderation. Finally, a SEM was built (Fig. 2) to test whether some of the effects of independent variables were mediated by parental physical activity. Mediators are variables being influenced by an independent variable and relaying part of this effect to a dependent variable. The SEM was estimated using the maximum likelihood method and included continuous and binary variables. The square root of the partial eta-squared statistic (i.e., the partial correlation between children’s MVPA and the respective independent variable after adjustment for all other covariates) was used to measure effect size. For the fit statistics of the SEM the “root mean squared error of approximation” (RMSEA), the “non-normed fit index” (NNFI) and the “comparative fit index” (CFI) were assessed. Model selection was based on the Akaike information criterion (AIC). The final model, contained the latent variable “parental PA behaviour” (PPAB = mediator) which was defined based on parent’s MVPA, their sport participation and their cycling during the measurement week. It provided estimates of the independent effects of age and sex of the child and the parents, the family setting and the environment on PPAB, and of the same variables and PPAB on MVPA of the child (dependent variable). The indirect effect is calculated by multiplying the association between the independent variable and parental physical activity behavior (a-path) with the association between PPAB and children’s MVPA (b-path). The direct effect describes the association between the independent variable and children’s MVPA adjusted for the parental physical activity behavior. Factor analysis was used to summarize single determinants into fewer representative factors (e.g. parental education and nationality). Because of the low power of children from the Italian speaking part in the SEM they were combined with the children from the French speaking part. Again, the model was re-run using parental MVPA based on 10 min bouts. The level of statistical significance was set at 0.05. Missing data were imputed by a separate category for categorical variables and by the mean for ordinal and continuous variables. Participants with missing dependent variables (MVPA for the comparison of objective measures and in additions parental cycling and parental sport participation in the SEM) were excluded from analyses. All analyses were conducted with STATA 14.0 [34].

**Table 1:** Characteristics and physical activity of the children

		N (%)	MVPA^c^ mean (standard deviation)	*p*-Value for difference in MVPA^c^
Overall	All	889 (100.0)	82.0 (37.2)	
Basic characteristics child				
Age				
6 to 9 year olds (ref.)		378 (42.5)	108.5 (33.2)	
10 to 12 year olds		311 (35.0)	71.7 (26.3)	
13 to 16 year olds		200 (22.5)	48.0 (18.4	< 0.001
Sex				
Boys (ref.)		466 (52.4)	92.1(39.4)	
Girls		423 (47.6)	70.9 (31.0)	< 0.001
Overweight Child				
No		756 (88.4)	82.0 (37.1)	
Yes		99 (11.6)	80.8 (37.9)	0.60
Parental characteristics				
Participation parents				
Mother only (ref.)		516 (58.0)	83.6 (37.8)	
Father only		203 (22.8)	79.9 (34.2)	
Both parents		170 (19.1)	79.8 (38.6)	0.35
Age participating parent^a^				
40 and less		227 (25.8)	98.9 (38.7)	
41 to 50		573 (64.3)	77.6 (35.1)	
51 and more		76 (9.9)	66.2 (31.7)	< 0.001
Overweight participating parent^a^				
No		613 (70.5)	81.5 (36.8)	
Yes		258 (29.6)	82.7 (37.5)	0.79
Family characteristics				
Nationality				
Swiss (ref.)		623 (70.1)	82.4 (37.7)	
Dual citizenship		174 (19.6)	80.6 (35.5)	
Non-Swiss		92 (10.4)	81.9 (37.0)	0.94
Highest education parents				
Low (ref.)		21 (2.4)	76.2 (27.7)	
Medium		479 (54.0)	80.7 (36.9)	
High		387(43.6)	84.0 (37.9)	0.64
Workload participating parent^a^				
50% and less (ref.)		629 (71.6)	83.2 (36.9)	
51–75%		127(14.5)	80.4 (39.0)	
75% and more		123 (14.0)	77.4 (36.3)	0.18
Parent child relationship^b^				
Score of 75 and less (ref.)		315 (36.2)	81.2 (38.0)	
76 to 90		322 (37.0)	85.4 (36.9)	
91 and more		234 (26.9)	78.2 (36.2)	0.04
Siblings				
0 (ref.)		159 (18.4)	75.0 (37.3)	
1		419 (48.44)	80.5 (36.9)	
2 and more		220 (25.4)	88.5 (36.5)	< 0.001
Parental co-activity				
No regular co-activity (ref.)		722 (82.2)	81.0 (36.8))	
Once a week and more		167 (18.8)	86.5 (38.6)	0.12
PA role model				
Sport participation parental^a^				
Never/seldom (ref.)		223 (35.5)	82.9 (39.1)	
At least once a week		253 (28.9)	77.0 (33.4)	
Several times a week		400 (45.7)	84.5 (37.8))	0.08
Bike for transport in target week^a^				
No		517 (60.6)	80.0 (36.0)	
Yes		336 (39.4)	85.4 (38.7)	0.07
Environment				
Walkability				
Low		297 (33.4)	78.4 34.7)	
Medium		275 (30.9)	83.3 (39.5)	
High		317 (35.7)	84.2 (37.2)	0.17
Urbanicity				
Rural area		302 (34.0)	79.8 (34.2)	
Agglomeration		411 (46.2)	81.8 (38.1)	
Urban area		176 (19.8)	86.3 (39.6)	0.24
Language region				
German		671 (75.5)	83.9 (37.8)	
French		184 (20.7)	76.3 (34.5)	
Italian		34 (3.8)	76.1 (35.1)	0.03

^a^If both parents participated, answers from the mother were considered

^b^The parent child relationship is based on the family module of the KINDL^R^ questionnaire. The highest score of 100 denotes a perfect relationship and the lowest score of 1 denotes a very bad relationship

^c^MVPA = moderate to vigorous physical activity

Missing’s were substituted by an own category but not presented in the table

## Results

Figure 1 presents the study population flow chart. 3108 (74%) of the contacted children were interviewed by phone (first part of the study) and 1611 (78%) of the children invited for the second part of the study wore an accelerometer. 1320 children provided valid accelerometer data and for 889 of these children valid accelerometer data of at least one parent was also available. These children and adolescents did not significantly differ from the remaining 431 children providing valid accelerometer data with respect to sex and MVPA, but they were slightly younger (10.4 versus 11.0 years) and their parents were more likely to have a high education (43.6% compared to 33.4%).

Characteristics of the children and associated mean levels of accelerometer based MVPA are presented in Table 1. Mean age of the subjects participants was 10.4 (2.6 years) and slightly more boys (52.4%) participated in the study. Mean MVPA decreased with age and was significantly higher in boys than in girls. The decrease in children’s and adolescents’ MVPA with increasing parental age disappeared upon adjusting for the children’s age. Finally having siblings and living in the German part of Switzerland were associated with more MVPA.

Characteristics of mothers and fathers and associated mean levels of objectively measured total MVPA per day and of minutes spent in bouts of ≥10 min MVPA per day) are presented in Table 2. Of the participating parents, 686 (64.8%) were mothers and 373 (35.2%) were fathers. For 170 (19.1%) children, valid measurements of both parents were available. On average, accelerometer based MVPA was lower in both parents if they were overweight or did sport less than once a week. For mothers, MVPA decreased with a higher workload. Like their children, mothers of at least 2 children and living in the German speaking part of Switzerland were more active. Among mothers, but not fathers, self-reported bike use in the target week was positively associated with MVPA.

**Table 2:** Characteristics and physical activity of the parents

	Mothers Minutes/day of MVPA			Fathers Minutes/day of MVPA		
	N (%)	Total min mean ± (standard deviation)	≥10 min bouts mean (standard deviation)	N (%)	Total min mean (standard deviation)	≥10 min bouts mean (standard deviation)
Overall	686 (100.0)	37.1 (21.4)	16.1 (16.4)	373 (100.0)	42.8 (23.0)	15.3 (16.3)
Basic characteristics parent						
Age:						
40 and less	199 (29.4)	36.3 (21.6)	14.3 (15.5)	53 (14.6)	45.3 (27.6)	14.5 (14.0)
41 to 50	432 (63.9)	37.5 (21.7	17.1 (16.8)	238 (65.4)	43.4 (22.1)	16.2 (17.0)
51 and more	45 (6.6)	39.1 (18.7)	15.7 (16.2)	73 (20.1)	40.3(21.8)	13.4 (15.7)
Overweight parent						
No	524 (78.0)	38.1 (21.2)	16.7 (16.5)	168 (46.2)	47.2 (23.7)	19.1 (18.4)
Yes	148 (22.0)	33.9 (22.2)**	14.0 (16.2)***	196 (53.8)	39.1 (21.9)***	12.6 (13.8)
Characteristics of the child						
Age children						
6–9 years ref.)	301 (43.9)	36.8 (21.0)	15.3 (16.0)	144 (38.6)	42.4 (24.9)	14.8 (15.0)
10–12 year s	234 (34.1)	37.9 (21.1)	16.3 (15.0)	136 (36.5)	42.2(21.5)	14.5. (17.9)
13–16 year	151 (22.0)	36.7 (23.1)	17.1 (19.2)	93 (24.9)	44.2 (22.1)	18.0 (19.9)
Overweight child						
No	581(88.6)	36.7 (21.1)	15.8 (16.1)	313 (86.5)	42.1 (21.7)	15.4 (16.5)
Yes	75 (11.4)	39.3 (23.5)	17.1 (17.4)	49 (13.5)	47.0 (29.5)	15.6 (16.2)
Family characteristics						
Nationality						
Swiss	485 (70.7)	38.1 (22.3)	16.7 (17.1)	267 (71.6)	42.3 (22.2)	14.6 (15.0)
Dual citizenship	134 (19.5)	35.0 (18.4)	14.5 (14.5)	69 (18.5)	45.0 (25.2)	18.0 (19.9)
Non-Swiss	67 (9.8)	34.9 (20.5)	14.1 (13.9)	37 (9.9)	41.7 (24.4)	14.5 (17.9)
Highest education parents						
Low	18 (2.6)	32.7 (20.0)	10.9 (14.2)	9 (2.4)	44.2 (21.2)	13.3 (16.1)
Medium	374 (54.7)	36.8 (22.3)	16.4 (17.2)	189 (50.8)	43.8 (25.4)	14.7 (16.0)
High	292 (42.7)	38.0 (20.4	16.0 (15.5)	174 (46.8)	41.6 (20.2)	16.1 (16.8)
Workload participating parent						
50% and less	492 (74.35)	38.2 (21.6)	16.8 (16.5)	26 (7.1)	34.7 (24.2)	14.8 (18.6)
51–75%	97 (14.7)	35.6 (19.4)	15.4 (15.5)	15 (4.1)	33.3 (20.9)	12.9 (19.6)
75% and more	73 (11.6)	31.4 (19.8)**	11.1 (12.6)**	327 (88.9)	43.7 (22.7)	15.6 (16.0)
Parent child relationship^a^ (score):						
75 and less	236 (35.12)	35.2 (20.8)	14.2 (14.7)	146 (39.9)	41.1 (24.7)	15.0 (16.7)
76 to 90	248 (36.9)	38.0 (22.0)	16.7 (17.6)	133 (36.3)	42.5 (21.7)	14.9 (16.9)
91 and more	188 (28.0)	38.4 (21.3)	17.4 (16.4)	87 (23.8)	45.3 (21.5)	16.3 (14.6)
Parental co-activity						
No regular co-activity	548 (79.9)	37.9 (21.7)	16.0 (16.6)	296 (79.4)	43.2 (23.5)	15.6 (16.8)
Once a week and more	138 (20.1)	37.7 (20.4)	16.3 (15.4)	77 (20.6)	41.3 (20.7)	14.4 (14.3)
siblings						
0	127 (19.0)	32.9 (19.2)	13.9 (14.6)	72 (19.9)	41.8 (22.7)	17.3 (18.6)
1	315 (47.1)	38.7 (22.1)*	17.5 (17.6)	176 (48.8)	42.1 (23.3)	15.3 (16.4)
2 and more	227 (33.9)	37.1 (21.0)	15.1 (14.8)	113 (31.3)	44.1 (22.5)	14.0 (14.7)
PA-Role model						
Sport participating parent						
Never/seldom	165 (24.5)	29.8 (20.5)	10.1 (13.2)	113 (30.7)	38.8 (22.5)	9.7 (12.0)
At least once a week	195 (28.9)	32.6 (19.0)*	12.4 (13.3)**	97 (26.4)	38.0 (19.2)	11.6 (13.8)
Several times a week	314 (46.6)	43.7 (21.4)***	21.5 (18.0)***	158 (42.9)	48.5 (24.3) ***	21.9 (18.2)***
Bike for transport in target week^2^						
No	390 (59.8)	35.2 (21.3)	14.9 (16.1)	213 (59.0)	41.9 (22.4)	14.9 (16.9)
Yes	262 (40.2)	39.9 (21.7)**	17.8 (17.0)**	148 (41.0)	44.0 (23.9)	16.6 (15.7)
No regular co-activity	548 (79.9)	37.0 (21.7)	16.0 (16.6)	296 (79.4)	43.2 (23.5)	15.6 (16.8)
Once a week and more	138 (20.1)	37.7 (20.4)	16.3 (15.4)	77 (20.6)	41.3 (20.7)	14.4 (14.3)
Environment						
Walkability						
Low	229 (33.4)	35.3 (21.3)	14.8 (15.2	127 (34.1)	42.8 (25.1)	14.6 (15.4)
Medium	214 (31.2)	37.6 (21.9)	16.4 (16.5)	117 (31.4)	45.5 (22.9)	16.4 (16.1)
High	243 (35.4)	38.5 (21.1)	16.9 (17.3)	129 (34.6)	40.3 (20.5)	14.9 (17.3)
Urbanity						
Rural area	232 (33.8)	35.1 (20.8)	14.4 (14,9)	130 (34.9)	41.4 (22.7)	12.8 (13.4)
Agglomeration	316 (46.1)	37.7 (21.9)	16.9 (16.8)	169 (45.3)	43.3 (23.3)	16.3 (16.0)
Urban area	138 (20.1)	39.2 (21.3)	16.8 (17.7)	74 (19.8)	43.9 (22.8)	17.6 (18.9)
Language region						
German	521 (76.0)	38.5 (22.0)	17.0 (16.9)	266 (71.3)	43.3 (22.9)	15.8 (15.8)
French	140 (20.4)	32.3 (19.0)**	12.5 (13.9)**	97 (26.0)	41.4 (22.7)	13.5 (16.3)
Italian	25 (3.6)	35.1 (19.8)	16.0 (16.1)	10 (2.7)	42.0 (29.3)	20.4 (27.3)

*p < 0.05, **p < 0.01, ***p < 0.001

^a^The parent child relationship is based on the family module of the KINDL^R^ questionnaire. The highest score of 100 denotes a perfect relationship and the lowest score of 1 denotes a very bad relationship

^b^MVPA = moderate to vigorous physical activity

Missing’s were substituted by an own category but not presented in the table

Associations between the child’s and parent’s MVPA were generally stronger when considering the total active minutes compared to bouts of MVPA. However, the results were similar, therefore only results for parental total MVPA are presented in Table 3, while the association with parental bouts is shown in the Additional file 1. Parental MVPA was statistically significantly associated with their child’s MVPA although the effect was relatively small. The child’s MVPA tended to be more strongly associated with the MVPA of the mother than with the one of the father. An increase of 1 min of mothers and of fathers MVPA was associated with 0.24 and 0.21 more MVPA in children, respectively. An exception was the strong association between father and child MVPA in non-Swiss families and families living in Italian-speaking Switzerland (Table 3). But also the association between mothers’ and children’s MVPA was highest in Italian-speaking regions. Associations between parents’ and children’s PA were strongest for children aged 10 to 12. Some associations were modified by the sex of the parent: Father’s PA was only associated with that of their sons, but not with that of their daughters, whereas mothers’ PA showed similar associations with PA of sons and daughters. While the association between fathers’ and children’s PA was strongest if the father had a high educational level, if the parent-child relationship was good, if the child had two siblings or more, or if bikes for transport were used, such patterns were not found or even reversed in mother-child pairs. For example, stronger associations were observed in the group of mother-child pairs where the mother had lower educational levels.

**Table 3:** Association between children’s and parental log-transformed minutes spent in MVPA (from linear regression models)

	Mother:		Father:	
	Total minutes spent in MVPA^c^ Coeff. (95%CI)^a^	*p*-value for heterogenerity^b^	Total minutes spent in MVPA^c^ Coeff. (95%CI) ^a^	*p*-value for heterogenerity^b^
All	0.12 (0.8; 0.15)***		0.09 (− 0.04; 0.15)***	
Age child		0.04		0.31
6–9 years	0.06 (0.01; 010)*		0.04 (− 0.02; 0.18)	
10–12 years	0.15 (0.09; 0.21)***		0.12 (0.01; 0.23)*	
13–16 years	0.12 (0.04; 0.20) **		0.13 (− 0.01; 0.26)	
Sex		0.89		0.14
Boys	0.11 (0.06; 0.16)***		0.13 (0.06; 0.21)**	
Girls	0.12 (0.07; 0.17)***		0.05 (− 0.03; 0.13)	
Overweight child		0.70		0.96
No	0.11 (0.07; 0,15)***		0.05 (−0.05; 0.14)	
Yes	0.13 (0.01; 0.23)*		0.06 (−0.15; 0.28)	
Family characteristics				
Nationality		0.32		0.34
Swiss	0.12 (0.08; 0.16)***		0.08 (0.01; 0.14)*	
Double citizen	0.13 (0.04; 0.23)**		0.12 (−0.05; 0.29)	
Non-Swiss	0.06 (−0.05; 0.17)		0.24 (0.01; 0.48)*	
Education		0.13		0.15
Low	0.34 (0.09; 0.58)*		Less than 10 pairs of father-child measures	
Medium	0.11 (0.07; 0.16)***		0.07 (−0.00; 0.14)	
High	0.12 (0.07; 0.17)***		0.15 (0.06; 0.24)**	
Workload participating parent		0.40		0.98
< 50%	0.10 (0.06; 0.14)***		0.11 (−0.07; 0.29)	
51–75%	0.17 (0.07; 0.27)***		0.09 (−0.37; 0.54)	
> 75%	0.13 (0.04; 0.22)**		0.09 (0.03; 0.15)**	
Age parent		0.01		0.14
≤ 40 years	0.05 (−0.01; 0.11)		0.05 (−0.07; 0.17)	
41–50 years	0.13 (0.09; 0.17)***		0.08 (0.01; 0.16)*	
≥ 51 years	0.29 (0.10; 0.48)**		0.23 (0.08; 0.37)**	
Parent-child relationship score		0.46		0.78
Lowest tertile	0.15 (0.08; 0.21)***		0.08 (−0.01; 0.17)	
Medium tertile	0.10 (0.05; 0.15)***		0.06 (−0.03; 0.15)	
Highest tertile	0.09 (0.03; 0.15)*		0.12 (−0.02; 0.26)	
siblings		0.49		0.19
0	0.18 (0.09; 0.26)***		0.09 (−0.05; 0.22)	
1	0.12 (0.07; 0.17)***		0.05 (−0.03; 0.13)	
2 and more	0.09 (0.02; 016)**		0.14 (0.04; 0.24)**	
Overweight parent		0.48		0.21
No	0.12 (0.08; 0.16)***		0.14 (0.05; 0.23)**	
Yes	0.10 (0.03; 0.16)**		0.07 (−0.01; 0.14)	
Parental co-activity		0.07		0.22
No regular coactivity	0.11 (0.07; 0.14)***		0.08 (0.02; 0.14)**	
Once a week or more	0.18 (0.11; 0.26)***		0.17 (0.04; 0.30)**	
Role model				
Sport participation parent		0.52		0.54
Never/seldom	0.13 (0.06; 0.20)***		0.07 (− 0.03; 0.16)	
At least once a week	0.08 (0.01; 0.14)*		0.15 (0.02; 0.28)*	
Several times a week	0.10 (0.04; 0 l17)**		0.07 (−0.02; 0.16)	
Bike for transport in target week		0.93		0.35
No	0.12 (0.07; 0.16)***		0.08 (0.00; 0.15)*	
Yes	0.12 (0.06; 0.18)***		0.13 (0.04; 0.22)**	
Environment				
Walkability		0.86		0.42
Low	0.10 (0.05; 0.16)***		0.08 (−0.01; 0.17)	
Medium	0.13 (0.06; 0.19)***		0.15 (0.04; 0.27)**	
High	0.12 (0.06; 0.18)***		0.05 (−0.05; 0.15)	
Urbanity		0.79		0.85
Rural area	0.13 (0.08; 0.19)***		0.08 (−0.02; 0.17)	
Agglomeration	0.11 (0.06; 0.16)***		0.11 (0.03; 0.20)**	
Urban area	0.11 (0.02; 0.20)**		0.09 (−0.03; 0.22)	
Language region		0.58		0.00
German	0.11 (0.07; 0.15)***		0.08 (0.02; 0.14)**	
French	0.13 (0.06; 0.20)***		0.05 (−0.07; 0.17)	
Italian	0.25 (−0.09; 0.59)		0.64 (0.00; 1.28)*	

* < 0.05 ** < 0.01 *** < 0.001;

^a^Adjusted for age, age^2^ and sex of the child, season accelerometer type and accelerometer time

^b^Differences in effect estimates across strata were assessed by a chi2-test for heterogeneity. The presence of significant heterogeneity was interpreted as indicating moderation

^c^MVPA = moderate to vigorous physical activity

Each coefficient represents the association between a parent’s and their child’s physical activity (within strata)

Missing’s were substituted by an own category but not presented in the table

As expected in the light of the interdependence of some of the social, family or environmental factors presented in Table 3, several variables loaded on the same factor: workload (reverse coding) and sibling (factor 1); parental education and nationality (factor 2); walkability and urbanity (factor 3). Along with other predictor variables, these factors were used in the SEM model including the latent variable “parental PA behaviour” (PPAB) as common contributory cause of parental MVPA, sports participation and cycling activity. The structure of this model is depicted in Fig. 2 and its results are presented in the Additional file 2. For the SEM 27 pairs of children and parents had to be excluded because of missing information about parental cycling or parental sport participation. PPAB was a strong mediator and determinant of offspring’s PA. It was significantly positively associated with the physical environment of the child (walkability and urbanity). The indirect effect of being an overweight parent (BMI ≥ 25) mediated by PPAB was negative. Yet, the effect on the direct pathway was positive resulting in a similar total effect on children from overweight and non-overweight parents Table 4).

**Table 4:** Total, direct and indirect effects on children’s moderate to vigorous physical activity (MVPA)

Independent variables:	Direct effect on children’s MVPA (in min/day) (c-path)	Indirect effect on children’s MVPA (mediated by parental physical activity behaviour) (in min/day) (ab-path)	Total effect on children’s MVPA (in min/day)	Size of the effect on children’s MVPA^a^
Characteristics child				
Age child (youngest = ref.)	−30.1	–	−30.1	0.66
Sex child (m = ref.)	−21.9	–	−21.9	0.40
Overweight (no = ref.)	−5.5	–	− 5.5	0.07
Parental physical activity behaviour (PPAB)	–	7.1	7.1	0.18
Parental characteristics				
Age parent (youngest tertile = ref.)	−4.1	0.1*7.1 = 0.7	−3.4	0.08
Sex parent (m = ref.))	1.9	−0.4*7.1. = −2.8	− 0.9	0.03
Overweight parent (no = ref.)	7.1	−0.6*7.1 = −-4.3	2.8	0.11
Family characteristics				
Workload and siblings (factor 1)	1.2	0.0*7.1 = 0.0	1.2	0.043
Sociocultural family environment (nationality and education of the parents = factor 2)	−0.1	−0.1*7.1. = − 0.7	−0.8	0.01
Co-activity (yes = ref.)	0.2	0.2*7.1 = 1.4	1.6	0.00
Parent-child relationship (lowest tertile = ref.)	0.7	0.1*7.1 = 0.7	1.4	0.02
Environment				
Walkability & Urbanity (factor 3)	0.7	0.1*7.1. = 0.7	1.4	0.03
Language (German = ref.)	−5.2	−0.2*7.1 = −1.4	6.6	0.08

The total effect = indirect effect (via parental physical activity behavior) + direct effect.

The indirect effect is calculated by multiplying the association between the independent variable and parental physical activity behavior (a-path) with the association between parental physical activity behavior and children’s MVPA (b-path)

The direct effect describes the association between the independent variable and children’s MVPA adjusted for the parental physical activity behavior (see also Fig. 2)

MVPA = moderate to vigorous physical activity, PPAB = parental physical activity behavior

^a^Defined as square root of eta squared (i.e., the partial correlation between MVPA of the child and the respective variable, after adjustment for all other variables)

Such a compensating direct effect is missing for the “sociocultural environment” (factor 2) and for living in the non-German-speaking part of Switzerland where both the direct and indirect effects were negative. The same SEM-model for MVPA bouts showed largely similar results (data not shown).

## Discussion

According to objective measures in the nationwide SOPHYA study, parental modelling had an impact on children’s MVPA but some differences in the association by sociodemographics and environmental factors were identified. The pathway analysis in our SEM for MVPA of the child, including a latent variable reflecting parents’ physical activity behaviour (PPAB), showed direct effects (child age, sex, and overweight, parental overweight and language region) and indirect effects mediated by PPAB (parental sex and overweight, the sociocultural environment, the physical environment and the language region). This is in line with ecological models [6] positing that multiple levels of influence determine behaviour and that these interact across levels including intrapersonal factors such as age and sex [35], which were the strongest determinants of children’s MVPA in our model.

In the present analysis an increase of mothers and fathers MVPA of one minute was associated with an increase of 0.24 and 0.21 min respectively. At first glance this appears a small effect. However, a mere increase of 15 min per day has been shown to meaningfully reduce a cardiovascular risk score in children [36] pointing to a health enhancing potential for later life. Therefore, interventions to increase children’s PA should not only focus on children but also on parental physical activity. Other studies testing the association between parental and children’s PA showed mixed results [10–12], presumably due to the use of different methods (objective measures vs. self-report) [12], different age-groups [12, 14] and different outcomes (MVPA, total PA, number of daily steps, meeting the guidelines) [12, 14, 15], with a tendency of stronger associations when using objective measures [12]. When comparing parental and children’s PA, the question of whether we should use adult bouts of physical activity or every single minute of MVPA has not been discussed so far. Using 10 min bouts in adults is in accordance with the WHO guidelines [37] but does not suit children’s PA behaviour, which typically consists more of short intermittent activity bouts [38]. In our sample, associations were stronger, when using all minutes of MVPA of the parents indicating that role modelling might also include shorter activities such as taking the stairs instead of an elevator.

The examination of moderators (e.g. age, sex, parental education, sociocultural background) and contextual factors (e.g. family environment, neighbourhood walkability) is important. It can provide guidance for the adaptation of effective PA interventions to specific target groups [15, 16]. Previous studies criticized the limited assessment of such moderators [16, 19] or the restriction of studies to narrow age-groups [14]. In the SOPHYA study a national sample of school-aged children of different ages covering also the critical transition from childhood into adolescence was assessed and several intrapersonal, interpersonal and environmental moderators were included. In adolescents, the influence of parental modelling is expected to give way to the emerging influence of peers. However, the fact that the association between parental PA and adolescents’ PA remained suggests a potential sustained effect of role modelling at a younger age through the establishment of a social norm regarding PA later in life or a persistent influence of parents’ PA behaviour even in adolescents [11, 39]. Future studies should assess the age-specific impact of parental modelling taking into account parental support and parental attitudes towards sport. In addition, it would be of interest to assess, how parental modelling persists against the influence from peers.

A recent meta-analysis including both studies with objective PA-measurements and with self-reports, found that parents PA influenced activity patterns in boys but not girls [12]. In the SOPYHA study this was only true for fathers whereas mother’s PA was associated with PA of their daughters and of their sons. Yet, the modification of role modelling by the sex of the parent with regard to physical activity may further depend on the socio-cultural context. While role modelling of mothers was stronger compared to role modelling of fathers in Swiss families, only an association of MVPA between fathers and their children was observed in non-Swiss families. This finding highlights the importance of studying and testing physical activity interventions in different settings and to plan subgroup specific interventions.

A factor which increased the effect of parental modelling was “doing sport together at least once a week”. Unexpectedly and in contrast to previous studies [12] there was a tendency that the association between children’s and parents’ PA was lower if the mother-child relationship was strong. On the one hand this may suggest that a good relationship is also influenced by the time spent sedentary, e.g. talking together, reading a book out or playing a board game, which is not identified when comparing minutes spent in MVPA. On the other hand, very active children can also strain the parent-child relationship.

Overweight parents were significantly less active, but this did not result in a lower MVPA of their offspring. In the SEM model, we found a negative effect of parental overweight on the latent variable PPAB but this negative indirect pathway was compensated by a positive direct pathway resulting in a similar total effect (Fig. 2 and Table 4). This compensating effect on the direct pathway can occur by role modelling of the other parent; furthermore parents can influence their children also via parental support without being active themselves [11]. Finally the social environment can also motivate children to adopt an active lifestyle [40] and compensate the negative influence via the PPAB (indirect pathway). Such type of a social environment might be missing in the French and the Italian speaking areas, where PPAB seems to be less strong and a compensating effect via the direct pathway is missing. It might be that regional clustering of lower PA leads to adopted social norms [40]. So far it is not clear what causes the different behaviours and attitudes across language regions. In Switzerland the sport policies cover all regions and previous studies showed that the built environment does not explain the regional differences in Swiss children’s PA [41]. In SOPHYA the walkability of the neighbourhood and the urbanicity had an impact on PPAB. However, the direction of causality cannot be derived from our data. While a favourably built environment can increase parental PA, active parents may also choose to live in an activity-friendly environment. For such analyses, longitudinal data is needed.

The impact of the family environment on children’s MVPA should not be reduced to the parents. One of the strongest associations of both children’s and also parent’s MVPA was with the presence of siblings. Also co-activity with other peers has an important effect [42], but this was not assessed in the current study.

The strength of the present study includes the use of objective measurements of PA in a large sample, the avoidance of clusters by schools or neighbourhood areas by using register data for the recruitment of the children and the inclusion of moderators from different levels of a commonly used ecological model [6] which were shown to influence health behaviour independently, but also by interacting across levels [43]. However, some limitations should also be acknowledged. First, besides coactivity no pathway for parental support was included as the study focused solely on the role modelling part defined as the association between children’s and parents’ MVPA [12]. Second, the study had a cross-sectional design which makes it unsuited to infer causality. Finally, selection bias could have influenced the results. On the other hand, the SOPHYA study stands out for its remarkably high participation rate for a study conducted in a nation-wide context and including valid accelerometer data of both children and their parents.

## Conclusions

According to the results of the SOPHYA-study, parental modelling might be a determinant of children’s PA. It further appears that unfavourable parental modelling might be compensated by other factors like positive social norms. These social norms might be different in the French and Italian language region resulting in a less favourable parental PA modelling and in significant lower MVPA in both children and adolescents. In order to increase PA in children, multilevel interventions including policies, structural changes and changes of parental PA behaviour may be worthwhile strategies but would have to be age- sex and region- specific.

## Additional files

Additional file 1: **Table S1. **Relation between children’s MVPA and parental MVPA in bouts of ≥10 min (linear regression models). (DOCX 21 kb)

Additional file 2: Comparison of children with and without valid parental measurements. (DOCX 15 kb)

## Abbreviations

AIC: Akaike information criterion
CATI: Computer-aided telephone interviews
GIS: Geographic information system
MVPA: Moderate to vigorous physical activity
PA: Physical activity
PPAB: Parental physical activity behaviour
SEM: Structural equation model
SES: Socioeconomic status
SOPHYA: Swiss children’s Objectively measured PHYsical Activity
WHO: World Health Organisation
