# Supplementary material for: Objectively measured physical activity in population-representative parent-child pairs: parental modelling matters and is context-specific
Source: BMC Public Health. 2018 Aug 17;18:1024. doi: 10.1186/s12889-018-5949-9 (PMC6098593; doi:10.1186/s12889-018-5949-9)
Supplement: Supplementary file 1 — Table S1. Relation between children’s MVPA and parental MVPA in bouts of ≥10 min (linear regression models). (DOCX 21 kb) [file 12889_2018_5949_MOESM1_ESM.docx]

**Additional file 1: Table S1.** Relation between children’s MVPA and parental MVPA in bouts of ≥10 minutes (linear regression models)

|  | **Mother** | | **Father** | |  |
| --- | --- | --- | --- | --- | --- |
|  | **MVPA^c^ bouts of 10 minutes**  Coeff. (95%CI)^a^ | p-value^b^ for moderation | **MVPA^c^ bouts of 10**  **minutes**  Coeff. (95%CI)^a^ | p-value^b^ for moderation | |
| All | **0.03 (0.01; 0.04)***** |  | 0.01 (-0.01; 0.03) |  |  |
| Age child |  | **0.01** |  | 0.24 |  |
| 6- 9 years | 0.01 (-0.01; 0.02) |  | -0.00 (-0.02; 0.02) |  |  |
| 10-12 years | **0.05 (0.03; 0.08)***** |  | 0.03 (-0.00; 0.06) |  |  |
| 13- 16 years | 0.03 (-0.00; 0.06) |  | -0.01 (-0.05; 0.03) |  |  |
| Sex |  | 0.38 |  | 0.31 |  |
| Boys | 0.02 (0.00; 0.04)* |  | 0.02 (-0.01; 0.04) |  |  |
| Girls | **0.03 (0.02; 0.05)***** |  | -0.00 (-0.03; 0.02) |  |  |
| Overweight child |  | 0.89 |  | 0.98 |  |
| No | **0.03 (0.01; 0.04)***** |  | 0.01 (-0.01: 0.03) |  |  |
| Yes | 0.02 (-0.02; 0.07) |  | 0.01 (-0.03; 0.05) |  |  |
|  |  |  |  |  |  |
| **Family characteristics** |  |  |  |  |  |
| Nationality |  | 0.59 |  | 0.95 |  |
| Swiss | **0.03 (0.02; 0.05)***** |  | 0.01 (-0.01; 0.02) |  |  |
| Double citizen | 0.02 (-0.02; 0.05) |  | 0.01 (-0.04; 0.05) |  |  |
| Non-Swiss | -0.00 (-0.05; 0.04) |  | 0.02 (-0.05; 0.09) |  |  |
| Education |  | 0.87 |  | 0.56 |  |
| Low | 0.05 (-0.08; 0.18) |  | Less than 10 pairs of  father-child measures |  |  |
| Medium | **0.03 (0.01; 0.05)***** |  | 0.00 (-0.02; 0.03) |  |  |
| High | **0.03 (0.01; 0.05)**** |  | 0.02 (-0.01; 0.04) |  |  |
| Workload participating parent |  | 0.23 |  | 0.60 |  |
| <50% | **0.02 (0.01; 0.04)**** |  | 0.01 (-0.06; 0.08) |  |  |
| 51-75% | **0.05 (0.02; 0.09)**** |  | -0.03 (-0.13; 0.07) |  |  |
| >75% | 0.02 (-0.01; 0.05) |  | 0.01 (-0.01; 0.03) |  |  |
| Age parent |  | **0.00** |  | 038 |  |
| ≤ 40 years | -0.01 (-0.03; 0.01) |  | -0.00 (-0.04; 0.03) |  |  |
| 41-50 years | **0.03 (0.02; 0.05)***** |  | 0.00 (-0.02; 0.03) |  |  |
| ≥51 years | **0.11 (0.04; 0.19)**** |  | 0.03 (-0.01; 0.07) |  |  |
| Parent-child relationship score |  | 0.92 |  | 0.36 |  |
| Lowest tertile | **0.03 (0.01; 0.05)**** |  | 0.00 (-0.03; 0.03) |  |  |
| Medium tertile | **0.02 (0.00; 0.04)*** |  | 0.02 (-0.00; 0.05) |  |  |
| Highest tertile | 0.03 (-0.00; 0.05) |  | -0.01 (-0.05; 0.03) |  |  |
| siblings |  | 0.98 |  | **0.01** |  |
| 0 | **0.05 (0.02; 0.08)**** |  | 0.01 (-0.03; 0.05) |  |  |
| 1 | **0.02 (0.01; 0.04)**** |  | -0.01 (-0.03; 0.02) |  |  |
| 2 and more | **0.02 (0.00; 0.05)*** |  | **0.03 (0.00; 0.07)*** |  |  |
| Overweight parent |  | 0.35 |  | **0.04** |  |
| No | **0.03 (0.02; 0.05)***** |  | **0.03 (0.00; 0.05)*** |  |  |
| Yes | 0.02 (.-0.01; 0.04) |  | -0.01 (-0.03; 0.01) |  |  |
| **Role model** |  |  |  |  |  |
| Sport participation parent |  | 0.71 |  | 0.65 |  |
| Never/seldom | 0.02 (-0.01; 0.04) |  | 0.01 (-0.02; 0.04) |  |  |
| At least once a week | 0.02 (-0.01; 0.04) |  | -0.01 (-0.04; 0.03) |  |  |
| Several times a week | **0.03 (0.01; 0.06)*** |  | 0.00 (-0.03; 0.04) |  |  |
| Bike for transport in target week |  | 0.48 |  | 0.95 |  |
| No | **0.02 (0.01; 0.04**** |  | 0.01 (-0.01; 0.03) |  |  |
| Yes | **0.03 (0.01; 0.06)***** |  | 0.01 (-0.02; 0.04) |  |  |
| Parental co-activity |  | 0.09 |  | 0.35 |  |
| No regular coactivity | **0.02 (0.01; 0.04)***** |  | 0.01 (-0.01; 0.03) |  |  |
| Once a week or more | **0.05 (0.02; 0.08)***** |  | 0.03 (-0.01; 0.06) |  |  |
| **Environment** |  |  |  |  |  |
| Walkability |  | 0.28 |  | 0.80 |  |
| Low | **0.02 (0.00; 0.05)*** |  | 0.01 (-0.02; 0.04) |  |  |
| Medium | **0.04 (0.02; 0.06)***** |  | 0.01 (-0.02; 0.04) |  |  |
| High | 0.02 (-0.00; 0.04) |  | -0.00 (-0.03; 0.03) |  |  |
| Urbanity |  | 0.86 |  | 0.91 |  |
| Rural area | **0.03 (0.01; 0.05)**** |  | 0.01 (-0.02; 0.04) |  |  |
| Agglomeration | **0.03 (0.01; 0.05)**** |  | 0.01 (-0.02; 0.03) |  |  |
| Urban area | 0.02 (-0.01; 0.05) |  | 0.02 (-0.02; 0.06) |  |  |
| Language region |  | 0.42 |  | **0.04** |  |
| German | **0.02 (0.01; 0.04)***** |  | -0.00 (-0.02; 0.02) |  |  |
| French | **0.04 (0.02; 0.07)**** |  | 0.02 (-0.02; 0.05) |  |  |
| Italian | 0.05 (-0.05; 0.15) |  | 0.20 (-0.16; 0.56) |  |  |

^a^ Adjusted for age, age^2^ and sex, season accelerometer type and measurement time

^b^ Differences by strata (moderation) were tested by a chi2-test for heterogeneity

^c^ MVPA = moderate to vigorous physical activity

*<0.05 ** <0.01 *** <0.001;

Each coefficient represents the association between a parent's and their child's physical activity (within strata).

Missings were substituted by an own category but not presented in the table
