# Supplementary material for: Objectively measured physical activity in population-representative parent-child pairs: parental modelling matters and is context-specific
Source: BMC Public Health. 2018 Aug 17;18:1024. doi: 10.1186/s12889-018-5949-9 (PMC6098593; doi:10.1186/s12889-018-5949-9)
Supplement: Supplementary file 2 — Comparison of children with and without valid parental measurements. (DOCX 15 kb) [file 12889_2018_5949_MOESM2_ESM.docx]

**Additional file 2: Table S2.** Comparison of children with and without valid parental measurements

|  | All children with accelerometer data  n(%) | Children without parental measurements  n(%) | Children with parental meauresment (included sample)  n(%) | Chi2 |
| --- | --- | --- | --- | --- |
| **Overall** | 1320 (100%) | 431 (100%) | 889 (100%) |  |
|  |  |  |  |  |
| **Age** |  |  |  |  |
| 6 to 9 year olds | 518 (39.2) | 140 (32.5) | 378 (42.5) |  |
| 10 to 12 year olds | 469 (35.5) | 158 (36.7) | 311 (35.0) |  |
| 13 to 16 year olds | 333 (25.2) | 133 (30.9) | 200 (22.5) | <0.001 |
|  |  |  |  |  |
| **Sex** |  |  |  |  |
| Boy | 678 (51.3) | 212 (49.2) | 466 (52.4) |  |
| Girls | 642 (48.7) | 219 (50.8) | 423 (47.6) | 0.3 |
|  |  |  |  |  |
| **Highest education parents** |  |  |  |  |
| Low | 51 (3.9) | 30 (7.0) | 21 (2.4) |  |
| Medium | 732 (55.7) | 253 (58.7) | 479 (53.9) |  |
| High | 530 (40.3) | 143 (33.2) | 387 (43.5) |  |
| No indication | 7 (0.2) | 5 (1.2) | 2 (0.2) | <0.001 |

The flow-chart of the study population showing how the sample was recruited and how representative it was, is presented in figure 1

If children or parents did not provide valid accelerometer data as described in the method part and if no information about age and sex were available, they were excluded from the sample. If only an item were missing, categorical variables were replaced by an own category and for ordinal and continuous variables single imputation was conducted.
